# Travelling Editorials

**Published:** 1843-07

**Authors:** 


THE WESTERN JOURNAL.

Vol. VIII.—No. I.

LOUISVILLE, JULY 1, 1843.

TRAVELLING EDITORIALS.

*Artificial Fountains.*

The most fertile part of South-Alabama, is destitute of permanent springs. This is especially true of the counties of Greene, Marengo, and Sumpter, with those of Lowndes, Noxubee, and Kemper in the adjoining parts of Mississippi. These counties lie on the opposite sides of the Tombeckbee and Tuscaloosa rivers, and embrace most of what is called the prairie and canebrake country—botanically different, but geologically almost identical. The soil is highly calcarious, and abundantly supplied with black mould. The substratum is, indeed, the disintegrated outcropping of a thick formation of marlite, which dips to the south. The deficiency of water within this fertile district was so great as to retard its settlement; but the Artesian auger has at length supplied the desideratum. A great many plantations have permanent wells, and the number is annually increasing. They vary in depth from one hundred and forty to more than five hundred feet. In general they are about three hundred. In a few of these borings, the water does not rise to the surface, but is conducted into a well dug by the side of the aperture. In the majority, however, it flows out, *pleno rivo*, and displays no variation in quantity or quality throughout the year. In making a voyage down the Tombeckbee (on whose narrow and winding way, overshadowed by lofty forests garnished with long moss, we are writing this paragraph), one discovers the geological mechanism on which these fountains depend. At Demopolis a grey, soft, almost cretaceous limestone, reaches from the surface of the plain on which the town is built, to the waters edge, and by the seams which its perpendicular face presents, the observer discovers, at a glance, that it dips to the south. At the distance of fifteen or twenty miles, the whole has disappeared beneath the river, and a different formation taken its place, to be succeded by others. Thus the dip of this tertiary formation is to the south, and the highlands, where the water-retaining strata crop out, are in the region where the Tombeckbee and Tuscaloosa have their origin. These facts explain why very deep borings, even eight and nine hundred feet, in the *southern* part of the district, have failed to bring up water. They encountered the soft limestone or marlite too far from its out-cropping—too remote from the highlands.

The water of all the Artesian-wells is substantially the same. It has a faint sulphurous taste and smell, almost absent in a few; and is so hard as to become white on the addition of acetate of lead. Still it is soft compared with the shallow wells, called *seaps* which are supplied by percolation from near the surface. We found the depth and temperature of eight of these fountains, in Greene county, to be as follows:

| | | | | |
|---|---|---|---|---|
| No. 1, | 112 feet, | | 66 of Fahr. | |
| " 2, | 255 | " | 68 | " |
| " 3, | 260 | " | 69 | " |
| " 4, | 290 | " | 67.5 | " |
| " 5, | 300 | " | 69 | " |
| " 6, | 307 | " | 69 | " |
| " 7, | 312 | " | 67.5 | " |
| " 8, | 320 | " | 67 | " |

To what shall we ascribe the difference of temperature of these fountains, when their depth is nearly the same? especially, how are we to account for number eight, with a depth of three hundred and twenty feet, being 2° colder than number three, the depth of which is only two hundred and sixty feet, or sixty feet less? This difference, according to observations on the temperature of the earth at different depths, should have made it a degree and a half colder than the other. It may be, that after the boring of a well has been finished, water of a higher level bursts in, and thus gives a temperature different from that of its bottom. It may be, also, that the water in reaching some of the wells, may have ascended from greater depths. Finally, some variety in the mineral character of the rocks below may exist, and produce a difference of temperature.

But to waive all speculation, we may say, that the temperature of these fountains affords additional evidence that the heat of the earth increases as we descend. For, first, their temperature is above the mean heat of the climate in this latitude; second, the shallowest of the whole is the coldest; third, we found the heat of a well eighty feet deep to be 63° and of a spring 62°, both in the same latitude with the wells.

How far do the facts afforded by the Artesian fountains sustain the conclusion from observations made elsewhere, that as we descend into the earth the heat increases at the rate of one degree for every forty-five feet? Let us see.

The average depth of the four deepest borings is three hundred and nine feet. If we deduct from this sum eighty-four feet, for the range of variable summer and winter temperature, we have a remainder of two hundred and twenty-five, or five times forty-five, which should give 5° degrees of increased heat. Let us take the difference between 62° and 63°, or 62.°5, as the heat at the point of constant annual temperature, and add to it five degrees, and we have 67.°5, as the temperature which the water issuing from the depth of three hundred and nine ought to possess, which is within six-tenths of a degree of the average heat of the four deepest wells. We may say, then, that the Artesian borings of Alabama give additional evidence, that as we descend into the earth its temperature increases at about the ratio of a degree for every forty-five feet.

Let us in conclusion say a word on the value of this discovery to people of the Artesian district. 1st. Although the water is warm and somewhat sulphurous, it is so much superior to what the operatives on the cotton plantations drank before, that a state of improved health has been already observed on some of them. 2d. An abundant supply of stock water has been obtained. 3d. Water for any degree of irrigation can be had in abundance, of a temperature well suited to that object. 4th. By boring with larger augers, and uniting the streams from several apertures, each farm may have a permanent water power, for grinding, sawing, working the cotton gin, the press, and all other purposes. Thus, science is more than supplying a natural deficiency; and entitling herself to the gratitude of a new community, who are far from cherishing for her any overweening admiration.

### *Contact of Primitive and Tertiary Rocks.*

At Wetumpka, foot of the rapids of the Coosa river, the tertiary formations rest directly on strata of mica-slate which dip to the N. N. W. at an estimated angle of 45°. The upheaving of this primitive formation has perhaps raised the tertiary, and given them the southern inclination of which we have spoken in the preceding article. The disintegration of this slate has liberated an immense quantity of mica, which sparkles in the adjoining soil, and issues with the water of its springs, which deposit it on standing. Some of the medical gentlemen of Wetumpka attribute to this micaceous impregnation of the water, a chronic diarrhœa, which prevails at that place much more than in other parts of the State.

### *Legalized Quackery.*

Alabama has long had a law denying to those who practise medicine without a diploma or a license, the benefit of her courts in the collection of their debts. We do not suppose it has done any more good in this than in other states; but still it served as a theoretical expression in favor of science. Lately, however, it has been so modified as not to interfere with "any persons" who practise on the "botanical system of *doctor* S. Thompson;" provided, nevertheless, that they "do not bleed, apply a blister of Spanish flies, administer calomel or any of the mercurial preparations, antimony, arsenic, tartar-emetic, opium, or laudanum!" It would be difficult to say whether *doctor* Thompson's patent, or this law, is the more precious specimen of empyricism. It is marvellous, that a people so enlightened as those of Alabama, should allow their statute book to be made ridiculous by such nonsense.

### *Mesmerism.*

Alabama is not more thoroughly overrun with the disciples of *doc-*

*tor* Thompson, than those of Dr. Mesmer. Anxious to have the phenomena of Mesmerism subjected to a rigid examination, the true separated from the false, and the public mind kept in a healthy condition in reference to both, we cannot but regret to see one travelling mountebank after another, traversing the south-west, for the purpose of extracting money from those who are credulous enough to believe, that *he* can do any thing in which men of sense will confide. It is truly unfortunate for Mesmerism that it should have fallen into such hands. Things are coming to that kind of pass, that we shall soon not be able to distinguish the pass of an impostor from the pass of a scientific Mesmerizer, and, in discouragement, pass the whole by; not even making it a pastime of a passable kind, as it was in days past; and when this happens, there is great danger that it will be passed over, and fall into a somnambulic state, from which the passes of the most scientific hand may not awaken it, till the remembrance of what is now doing to degrade it has past away.

*Small amount of operative Surgery in the South-West.*

Our brethren of Alabama and eastern Mississippi, represent that the number of surgical cases in their practice is very small, and we incline to the same conclusion. There are, according to the best observations we have been able to make, fewer cases of calculus, and diseases of the eye requiring surgical treatment, than in the west; and there may, for aught we know, be other differences in favor of the south-west. The chief reason, however, for the rarity of surgical cases is to be found in the pursuits of the people. In the miscellaneous agriculture of the west and its mechanical operations, accidents requiring the aid of the surgeon, are far more frequent than they can ever be in cotton fields; where none of a serious kind are likely to occur. When, however, injuries are inflicted in this country, death by tetanus follows, far oftener than in higher latitudes, and this is true not only of the black, but of the white population.

*Old Doctors.*

In the north and west every village has its old doctor—generally quite a respectable personage, with several peculiarities, and the self-complacency which comes from a life of usefulness, winding up amidst the regard of the neighborhood, and the respect of the younger physicians, who surround him. A sage of this kind is almost unknown in the south-west, except by title, which often indicates about as much clinical labor, as that of colonel testifies to military achievement. The reason of this difference between the south-west and the more northern portions of the Union, is three fold: 1st. A great many physicians die young; 2d. A number go to cotton planting; 3d. Not a few marry rich widows. Thus it is that causes the most opposite conspire to deprive this quarter of the benefits of ripe medical experience. How long the first will continue with its past energy, we cannot predict; but the second will soon cease unless the price of cotton should rise above five cents a pound; the last, however, is of a permanent character, for six or eight times as many husbands as wives die in this region. We shall expect the thanks of at least two classes of persons for this information.

### *Departure and Acknowledgments.*

In leaving the country watered by the Alabama, Coosa, Tallapoosa, Tuscaloosa, Tombeckbee, and other beautiful rivers, which in their confluence make up the Mobile, we may be permitted to say, that in forty days of active travel, we have every where met with the greatest hospitality; that our brethren have liberally and candidly poured out their experience; while the planters whom we have visited, have communicated many valuable facts. We have met with neither robbery nor insult; nor more than a dozen drunken men; nor one Bowie knife; nor seen a fist fight; nor heard six physicians backbite their brethren; nor encountered a drunken doctor—*nunc rara avis.* On the contrary we have been among a temperate, quiet and religious people, whose unvaried and unostentatious kindness demands from us this acknowledgment. D.

*Tombeckbee River,* June, 1843.

